# Positive Health and palliative care: an exploration among nurses

**DOI:** 10.1093/eurpub/ckac131.319

**Published:** 2022-10-25

**Authors:** C De Bot, J Dierx, M Echteld

**Affiliations:** 1 Researchgroup Living in Motion, Avans University of Applied Sciences, Breda, Netherlands; 2 Researchgroup End of Life Care, Avans University of Applied Sciences, Breda, Netherlands

## Abstract

**Background:**

The current WHO definition of health seems to no longer meet the changes in the current Dutch health system. An alternative approach which puts emphasis on health, not disease, is Positive Health (Huber et al, 2011). This focus shifts the emphasis on improving resilience and well-being rather than the perspective on what is lacking in health.In this study, the attitudes towards the concept of Positive Health of palliative care nurses are examined.

**Methods:**

A mixed-methods approach design was used for this study. This involved the collection of both quantitative and qualitative data. Among the nurses, 134 questionnaires were administered. SPSS was used to analyze these results. For this study, six interviews were conducted and analyzed through thematic coding.

**Results:**

The quantitative analysis shows that the respondents are look positively about the Positive health description. Nurses find it important that it emphasizes that someone is more than his illness. In addition, the emphasis is on personal control. However, the question is also raised whether every patient can handle this. Nurses find the most important dimension ‘quality of life'. This is followed by the dimension ‘mental well-being’ and ‘spiritual existential'. The dimension ‘daily functioning’ is found to be the least important. The qualitative analysis also shows that the concept of Positive Health is viewed positively. The concept is considered positive, because it covers several areas of health. However, it was also indicated that the concept is still too broad. However, all respondents found that the concept can be applied in practice.

**Conclusions:**

This study shows that palliative care nurses have a positive attitude towards Positive Health. Nurses consider the all the dimensions important and also embed the aspects of it in their daily practice. However, the implementation of new concept should be explored.

**Key messages:**

• Palliative care nurses have a positive attitude towards Positive Health.

• The implementation of Positive Health in palliative care should be explored.

